# Prevalence of Anemia in Children from Latin America and the Caribbean and Effectiveness of Nutritional Interventions: Systematic Review and Meta–Analysis

**DOI:** 10.3390/nu11010183

**Published:** 2019-01-16

**Authors:** Lucía Iglesias Vázquez, Edith Valera, Marcela Villalobos, Mónica Tous, Victoria Arija

**Affiliations:** Department of Preventive Medicine and Public Health, Faculty of Medicine and Health Science, Universitat Rovira i Virgili, 43201 Reus, Spain; lucia.iglesias@urv.cat (L.I.V.); edith.valera@estudiants.urv.cat (E.V.); marcela.villalobos@estudiants.urv.cat (M.V.); monica.tous@urv.cat (M.T.)

**Keywords:** anemia, school-age children, preschool children, Latin America, Caribbean, developing countries, low- and middle-income countries, meta-analysis

## Abstract

Anemia affects 1.62 billion people worldwide. Latin America and the Caribbean (LAC) comprise several developing countries where children are a population at risk. This systematic review and meta-analysis aimed to estimate the prevalence of anemia in this population. Electronic databases, reference lists, and websites of health ministries were searched until December 2018. Stratified analyses were performed using RevMan5.3 to estimate the overall prevalence of anemia in preschool and school-age children. The effectiveness of nutritional interventions was also evaluated. We included 61 studies from the 917 reviewed, which included 128,311 preschool- and 38,028 school-age children from 21 LAC countries. The number of anemic children was 32.93% and 17.49%, respectively, demonstrating a significant difference according to age (*p* < 0.01). No difference was observed by gender and only school-age children from low/very low socioeconomic status (SES) (25.75%) were more prone to anemia than those from middle SES (7.90%). It was not a concern in the Southern Cone but constituted a serious public health problem in the Latin Caribbean. Nutritional interventions reduced the prevalence from 45% to 25% (*p* < 0.01). Anemia is still a public health problem for children in LAC countries. National surveys should include school-age children. Further nutritional interventions are required to control anemia.

## 1. Introduction

According to the estimates of international organizations, about 1.62 billion people in the world suffer from anemia [1], which constitutes a global public health problem in both developing and industrialized countries. However, the prevalence of anemia is higher in developing areas where pregnant women, women of childbearing age, and young children are especially vulnerable [2]. Anemia has a multifactorial etiology and multiple factors frequently act simultaneously; in this regard, sociodemographic conditions have been strongly associated with the prevalence of anemia, especially in low income countries [2,3].

Anemia during childhood has been linked to growth delay, high risk of infections, and poor cognitive and motor development [2,4,5]. In addition, the long-term consequences of anemia in infancy may also compromise social interaction and work productivity later in life [2,6,7]. Therefore, anemia affects not only individual quality of life but also the social and economic development of the country, a particularly important issue in developing economies [2,7,8].

Latin America and the Caribbean (LAC) includes several countries with low or very low socioeconomic status (SES), one of the factors that most predisposes children to having a high risk of malnutrition and anemia despite the effort of some governments in the promotion of nutritional interventions [2]. According to a recent systematic review [9], the prevalence of anemia in children under 5 years old ranged in LAC countries from 7.6% in Costa Rica to 65% in Haiti. Regarding prevalence in school-age children, data are, however, scarce in the literature [4].

The knowledge of the prevalence of anemia and the associated sociodemographic conditions allow us to identify the main risk factors, which will be helpful to prioritize prevention strategies. Therefore, we present a systematic review and meta-analysis of the prevalence of anemia in preschool- and school-age children in LAC countries according to their age, gender, SES, and region where they live. The effectiveness of nutritional interventions on the prevalence of anemia was also evaluated.

## 2. Materials and Methods

### 2.1. Literature Search

The systematic review was undertaken following the Meta-analysis of Observational Studies in Epidemiology (MOOSE) guidelines [10]. The electronic databases PubMed, Scopus, and SciELO were searched independently by two authors (LIV and EV) up to December 2018 for studies that reported the prevalence of anemia in children from LAC countries. The search strategy used combinations of terms, including Medical Subject Headings, both in British English (listed here) and American spelling. These terms were anemia, iron deficiency, iron status, ferritin, child, childhood, preschool, school-age children, Latin America, South America and Caribbean. There were no restrictions in terms of language or year of publication. The reference lists of original studies and reviews were searched looking for additional studies of interest. We also searched for national surveys on health and nutritional status through the websites of health ministries, the Demographic and Health Surveys (DHS) Program (www.dhsprogram.com), the United Nations Children’s Fund (UNICEF) Multiple Indicator Cluster Surveys (MICS) program (http://mics.unicef.org/surveys), and the WHO Vitamin and Mineral Nutrition Information System (VMNIS) (http://www.who.int/vmnis/database/en/).

The titles and abstracts of the search results were assessed and the full-texts of the potentially relevant articles were read carefully. Those that met the following inclusion criteria were included: (a) observational studies or national surveys that report the prevalence of anemia in preschool children (under 5 years) and school-age children (6–12 years); (b) anemia defined according to the WHO indications, internationally recognized, or very close to them; (c) studies carried on in LAC; (d) national or representative studies (*n* ≥ 100), except for the meta-analysis on effectiveness of nutritional interventions that included all available literature regardless of the sample size. Case reports, comments, editorials, letters, reviews, systematic reviews and meta-analysis, and those studies that assessed children with some disease were excluded from the selection process.

To discuss the public health problem posed by anemia for children, some importance categories were defined, according to WHO recommendations, as follows: not a public health problem when prevalence was under 4.9%; mild public health problem for prevalence between 5 and 19.9%; moderate public health problem for prevalence between 20 and 39.9%, and severe public health problem when prevalence exceeded 40% [1].

### 2.2. Data Extraction and Quality Assessment

Data on the first author’s surname, year of publication, country, SES, study design, total sample size, number of children with anemia, prevalence of anemia, and 95% confidence interval (CI) of anemia were extracted from all the included studies. Prevalence of anemia and 95% CI before and after the nutritional interventions were also extracted for those articles that reported them. Data extraction was performed independently by LIV, MV, and EV.

Methodological quality of the studies was assessed using the Newcastle-Ottawa scale [11], following the recommendation of the Cochrane Non-Randomized Studies Methods Working Group. This scale categorizes the final score in good, fair, and poor quality according to three domains of the evaluated studies: selection, comparability, and outcome.

### 2.3. Data Analysis

The prevalence of anemia (%) and standard error (SE) reported by each study were used to obtain the overall prevalence of anemia in children. In studies where the SE was not reported, it was calculated based on the prevalence and sample size. When a study examined the same population at different ages, data from older children was included in the meta-analysis.

A random-effect meta-analysis was undertaken by using the Review Manager 5.3 software (The Cochrane Collaboration, Copenhagen, Denmark) by using the inverse-variance method. Heterogeneity was assessed by calculating I^2^ [12]. Results were visualized using forest plots and the potential publication bias using funnel plots. Sensitivity analyses were also conducted to assess the robustness of the results by evaluating whether they could have been markedly affected by a single study. Statistical significance was set at *p* < 0.05 for all the analyses.

Stratified analyses were performed by: age (preschool- vs school-age children), gender (boy vs girl), SES (middle vs low or very low), area of residence (urban vs rural), and LAC region, including Mexico, Central America, Latin Caribbean, Andean subregion, Brazil, Southern Cone and non-Latin Caribbean. As a complementary analysis, the stratification by gender was also applied to children between 9 and 12 years old under the hypothesis that girls could already menstruate at these ages, which could make a difference between boys and girls. Furthermore, a comparative analysis of the prevalence of anemia before and after the application of nutritional intervention programs was performed to evaluate their effectiveness. In this section, additional sub-analyses were done by selecting the interventions at the national or regional level separately.

## 3. Results

**Figure 1:**
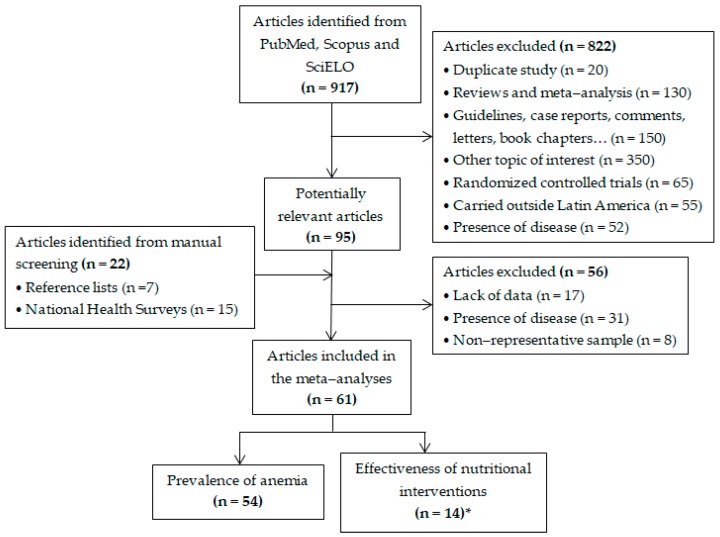
Flowchart of study selection. * This section includes seven new studies that were not present in the meta-analyses of prevalence of anemia.

The selection process of included studies is depicted in Figure 1. The search strategy performed in the PubMed, Scopus, and SciELO databases identified 917 publications that were firstly scrutinized by title and abstract; the full-texts of 95 articles were reviewed, of which 56 were excluded for the following reasons: children with any disease, lack of data on outcomes of interest, and non-representative sample was assessed. Twenty articles were additionally included to the selection process. Finally, the meta-analysis of prevalence of anemia included 54 studies with 128,311 preschool children and 38,028 school-age children from 21 LAC countries, evaluated between 1997 and 2018. The effectiveness of nutritional interventions was assessed in more than 6600 children from 10 LAC countries.

### 3.1. Study Characteristics

Table 1 and Table 2 present the information of the included studies for preschool and school-age children, respectively. Most of the studies used cross-sectional design, although one was a retrospective study [13], thirteen were national surveys about health, diet, and lifestyle [14,15,16,17,18,19,20,21,22,23,24,25,26], and five more were based on national surveys reports [27,28,29,30,31]. The studies were performed in Guyana [20], Argentina [29,32,33], Bolivia [26], Brazil [18,28,34,35,36,37,38,39,40,41,42,43,44,45,46,47,48,49,50], Chile [51,52], Colombia [27], Costa Rica [53,54], Cuba [55,56], Dominican Republic [24,25], Ecuador [16,57], Uruguay [58], Jamaica [59], Dominica [13], El Salvador [23], Guatemala [22], Haiti [17,60], Honduras [19], Mexico [30,31,61,62], Nicaragua [21], Panama [14], and Peru [15,63,64,65,66]. In relation to the SES, there were 25 studies conducted on middle income countries and the other 29 performed on low or very low-income countries. As to the methodological quality, good quality was reported in 34 studies and fair quality in the other 20 studies, according to Newcastle-Ottawa scale.

With regard to intervention programs designed to address the prevalence of anemia in the LAC countries, the meta-analysis evaluated 14 studies, seven of them exclusively included for that purpose. National plans were carried out in Costa Rica [53], Cuba [55], Dominican Republic (School Feeding Program) [25], Chile (National Complementary Feeding Program) [52], Ecuador (*Mi Papilla*) [67], and Mexico (*Oportunidades*) [61]. They implemented nutritional interventions and improvements in health services, especially targeting infants and school-age children. These national plans mostly arose between 1997 and 2000 and most of them routinely provided food fortified with micronutrients, including iron-supplemented formula milk, *papilla*, or snacks that children should consume at home. In addition, some of them had mandatory sessions on nutrition and health education aimed at mothers or those responsible for children [61]. In Peru, the effectiveness of supplementation with the multimicronutrient Chispitas^®^ in children under 3 years old was evaluated in two regions, Apurímac (*n* = 714) [65] and Ventanilla (*n* = 30) [68], after 6 months of intervention. Also in Peru, an educational and nutritional project [69] was implemented at the regional level between 2004 and 2007 for the prevention of anemia in children under 5 years of age. Finally, randomized controlled trials conducted in Bolivia [70], Haiti [60,71], Brazil [72], and Mexico [73] acted as short-term nutritional interventions based on iron fortification in preschool and school-age children.

### 3.2. Prevalence of Anemia in Children

**Figure 2:**
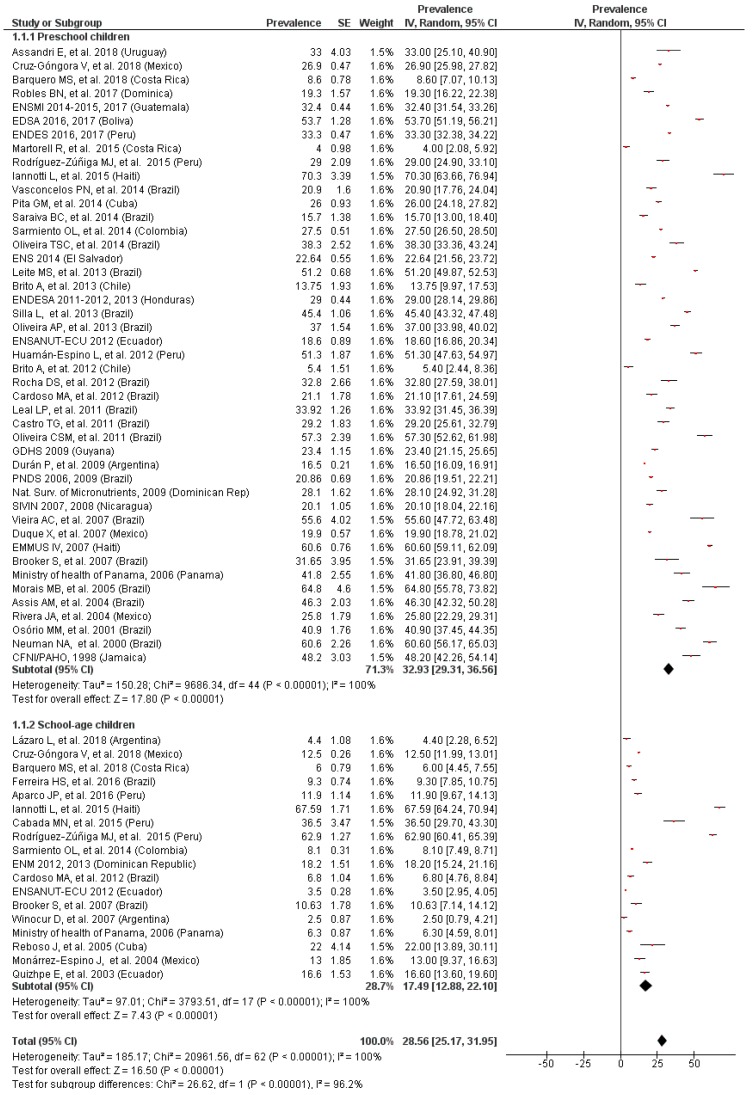
Prevalence of anemia by age.

After performing the systematic review, we observed that the prevalence of anemia ranged from 4% in Costa Rica [53] to 70.30% in Haiti [60] for preschool children and from 2.5% in Argentina [32] to 67.59% also in Haiti [60]. The overall prevalence of anemia in children under 12 years measured with a random effects model was 28.56% (95% CI: 25.17, 31.95), as it is shown in Figure 2.

### 3.3. Prevalence of Anemia by Age

The stratified analysis by age (Figure 2) highlighted that prevalence of anemia was statistically significant (*p* < 0.01) and higher in preschool children (32.93%; 95% CI: 29.31, 36.56) than in school-age children (17.49%; 95% CI: 12.88, 22.10). The heterogeneity was high (I^2^ = 96.2%). Visual inspection of the funnel plot showed that there was asymmetry, which shows the possibility of publication bias. The sensitivity analyses did not show any substantial variation in the overall results.

### 3.4. Prevalence of Anemia by Gender

**Figure 3:**
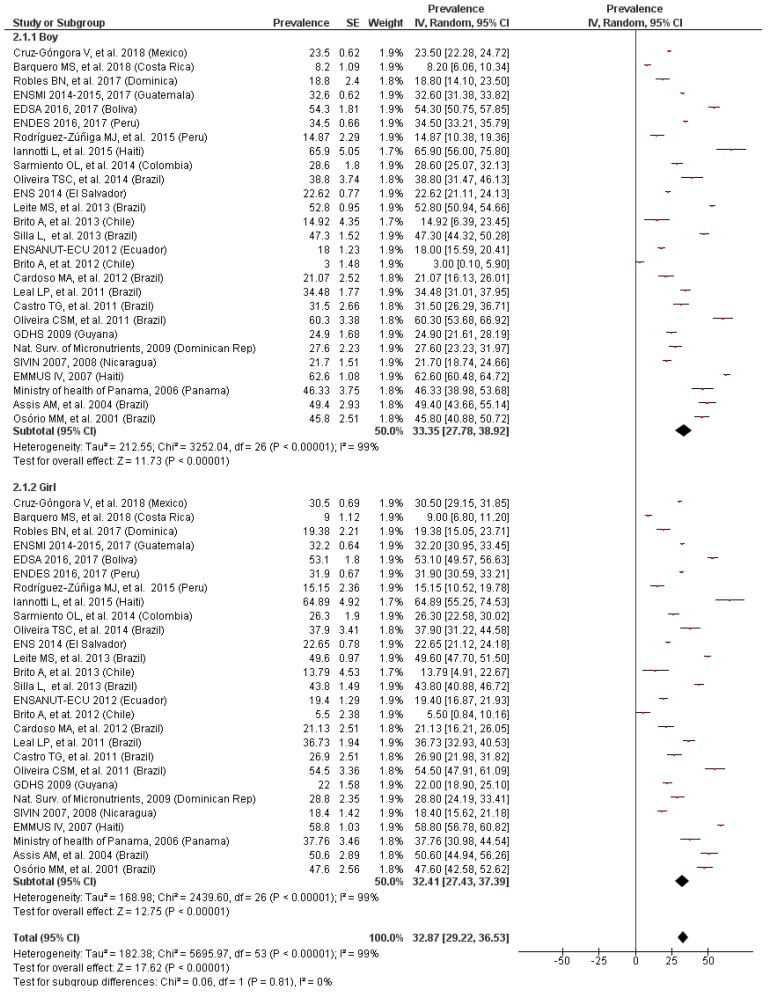
Prevalence of anemia in preschool children by gender.

**Figure 4:**
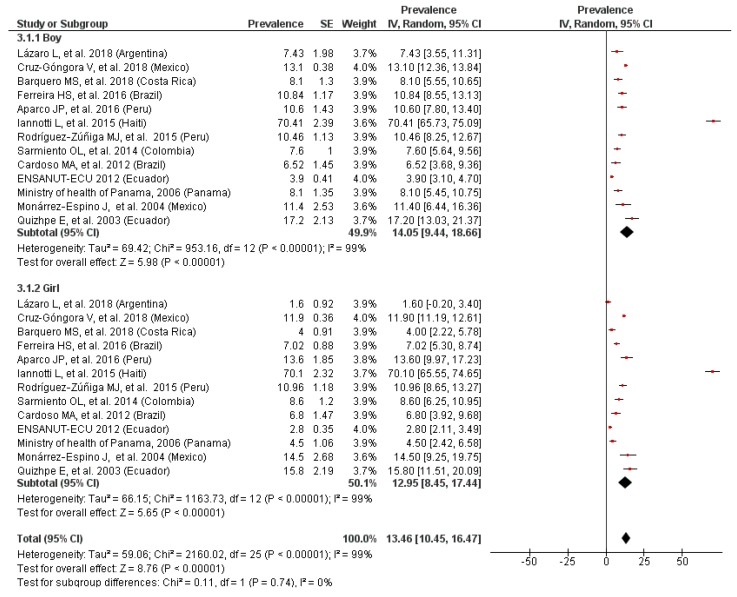
Prevalence of anemia in school-age children by gender.

According to the presented results, no differences were observed between boys and girls in the meta-analyses of the subsets of studies with data on gender for preschool- (Figure 3) or school-age children (Figure 4). The overall prevalence of anemia was, therefore, 33.35% and 14.05% for preschool and school-age boys, respectively, and a bit smaller for their female counterparts (32.41% and 12.95%, respectively).

The prevalence of anemia was also not different (*p* = 0.93) between boys (19.84%; 95%CI: 7.43, 32.25) and girls (19.07%; 95%CI: 6.71, 31.43) aged 9 to 12 years, evaluated in more than 3000 individuals (not shown).

The heterogeneity was null for these comparisons but possibly publication bias exists, visualized in an asymmetric funnel plot. The overall results did not change substantially after carrying out the sensitivity analyzes.

### 3.5. Prevalence of Anemia by SES

**Figure 5:**
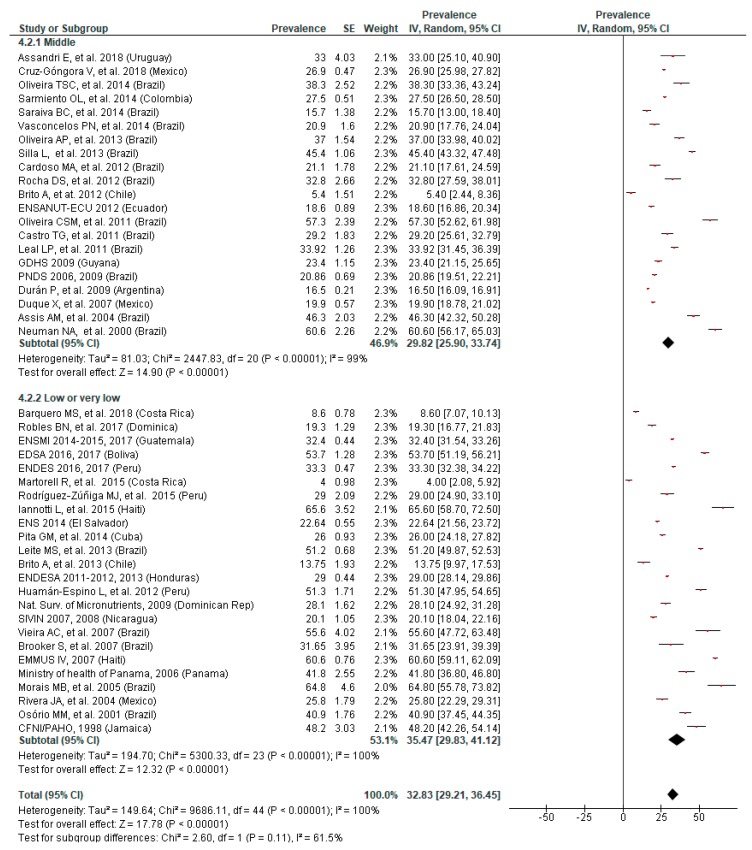
Prevalence of anemia in preschool children by socioeconomic status.

**Figure 6:**
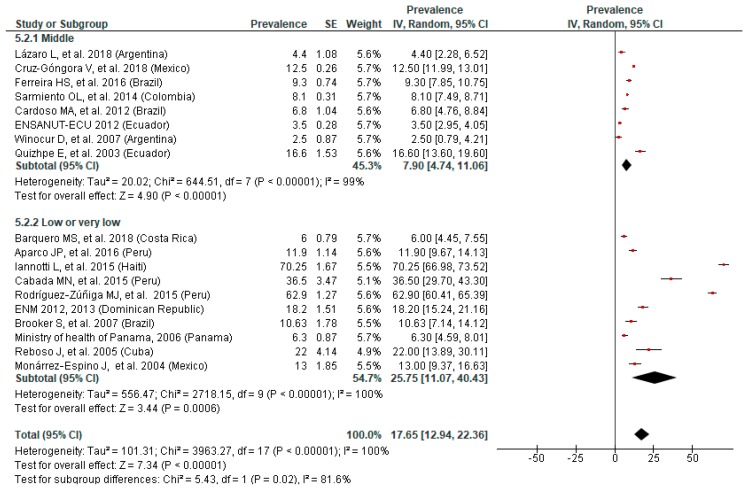
Prevalence of anemia in school-age children by socioeconomic status.

The subgroup analyses according to SES of children showed a statistically significant difference in the prevalence of anemia in school-age children (*p* = 0.02) but not in preschoolers (*p* = 0.11). However, for both preschool and school-age children, the overall prevalence of anemia was higher in low or very low SES areas (35.47% and 25.75%) than in those of middle SES (29.82% and 7.90%) (Figure 5 and Figure 6).

There was a fair and high heterogeneity (I^2^ = 61.5% and I^2^ = 81.6%) in the analysis of preschool and school-age children, respectively, and both funnel plots showed asymmetry, assuming publication bias. No substantial variation was noticed in the overall results following the sensitivity analyses.

### 3.6. Prevalence of Anemia by Area of Residence

No statistically significant differences were found in the prevalence of anemia in the stratified analysis according to the area of residence (rural or urban) in children of preschool age or school-age (*p* = 0.38 and *p* = 0.42, respectively). However, at both ages, prevalence was higher in rural than in urban areas: for preschool children, the percentages were 37.82% (95%CI: 30.43, 45.21) vs. 33.83% (95%CI: 28.79, 38.88), respectively, and for school-age children, 25.87% (95%CI: 6.50, 45.25) vs. 16.86% (95%CI: 6.93, 26.80), respectively.

The heterogeneity was null for these comparisons but possibly publication bias exists. The overall results did not change substantially after carrying out the sensitivity analyses (not shown).

### 3.7. Prevalence of Anemia by LAC Region

**Figure 7:**
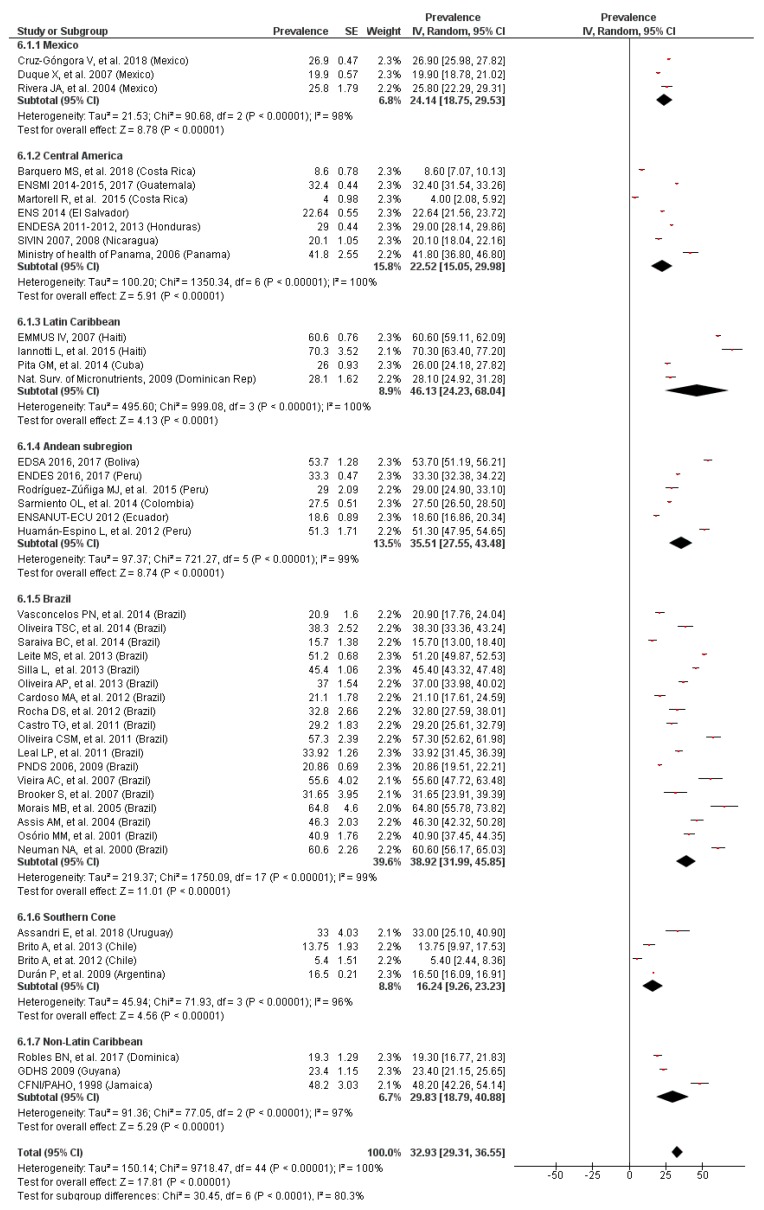
Prevalence of anemia in preschool children by LAC region.

**Figure 8:**
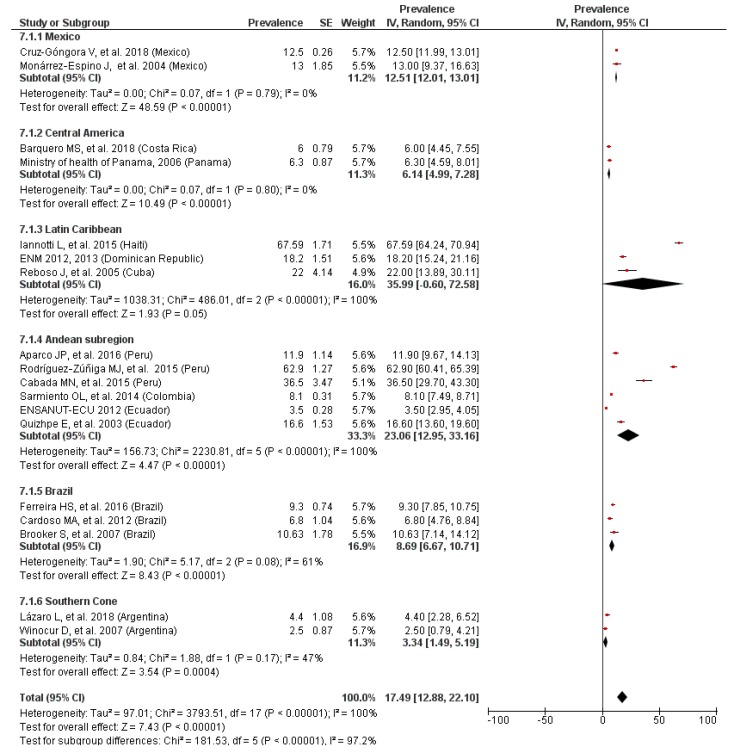
Prevalence of anemia in school-age children by LAC region.

A statistically significant difference (*p* < 0.01) in the prevalence of anemia was observed from the stratified analyses by LAC region (Figure 7 and Figure 8). The highest rate of anemia was in Latin Caribbean countries, while the Southern Cone region showed the lowest prevalence, regardless of the age of the children. The heterogeneity was high in both analyses and asymmetric funnel plots, suggesting that there was publication bias. No substantial variations in the overall results were shown when the sensitivity analyses were conducted.

### 3.8. Effectiveness of Iron Supplementation Programs

A subset of 14 articles evaluated the effectiveness of a nutritional intervention and reported data of the prevalence of anemia before and after its application. Iannotti et al. [60] in Haiti, reported data of preschool and school-age children separately. It is worth mentioning that in all cases children belonged to low or very low SES.

**Figure 9:**
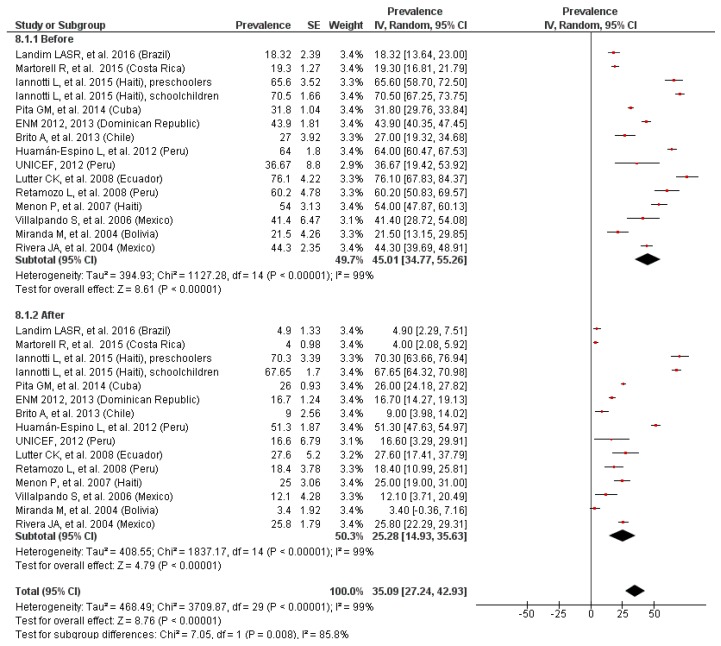
Effectiveness of nutritional intervention programs.

The results of the meta-analysis, shown in Figure 9, evidenced how the nutritional intervention reduced (*p* < 0.01) the prevalence of anemia from 45% to 25%. The heterogeneity was high (I^2^ = 85.8%) in this analysis. The funnel plot reported a possible publication bias. The sensitivity analysis did not show any significant variation in the overall results.

The evaluation of the effectiveness of the interventions carried out at a national level, which included more than 4500 children, showed a significant reduction (*p* < 0.01) in the prevalence of anemia with a decrease from 40% (95%CI: 29.02, 51.06) to 18% (95%CI: 8.87, 27.02). The heterogeneity was high for this comparison (I^2^ = 89.1%). On the contrary, the interventions carried out at the regional level or as randomized trials were not as successful (*p* = 0.14, I^2^ = 53.3%) (not shown).

## 4. Discussion

The present study incorporates several notable features. To date, knowledge about the prevalence of anemia in preschool and school-age children in LAC has been made up of isolated data for each country. We conducted a systematic review of the literature to gather the most up-to-date data available on the subject. Furthermore, we used meta-analysis to combine the findings from the identified studies and provide, for the first time, an overall estimate of the prevalence of anemia in more than 160,000 preschoolers and school-age children from 21 LAC countries, through the use of representative studies of fair and high quality, including national surveys.

Notwithstanding, some limitations should also be mentioned. First, moderate and high heterogeneity was observed in the stratified analyses by age, SES, LAC region, and in the meta-analysis of the effectiveness of the nutritional interventions. It could be due to the different number of studies included in each subgroup, variation in sample sizes and age of children among the studies, and the variability in their design. In addition, asymmetry was found in funnel plots of all meta-analyses, which could be attributed to the different methodological quality among the studies or to reporting bias such as publication, language, or citation bias. However, only the studies that followed the standardized diagnostic criteria for anemia, agreed by the WHO and internationally accepted for the studied populations, were included in the systematic review and meta-analyses, which guarantees certain comparability and reinforces the results. With respect to the effectiveness of nutritional interventions, the variability of the types of supplementation and food fortification applied in each case increases the dispersion of the results, undermining, in a certain way, the robustness of this meta-analysis. However, as discussed below, it gives a general idea of how the presence of some type of nutritional intervention acts in relation to the prevalence of anemia. This can be considered as a first step in the evaluation of public health policies related to nutrition to achieve increasingly successful interventions.

According to our findings, anemia is still a mild or moderate public health problem in most LAC countries, especially in children. In some countries, it is even a severe public health problem. Although the overall prevalence of anemia in children from LAC is 28.56%, this percentage conceals very different realities, ranging from 3.5% and 4% of anemia in Ecuador and Costa Rica, respectively, to 70% in Haiti. We describe the prevalence of anemia according to sociodemographic conditions and these analyses have allowed us to reveal that pre-school children, especially those from the Caribbean, the Andean subregion, and Brazil, are the population with the highest risk of anemia in LAC. However, children between 5 and 12 years old are not exempt from risk either, especially those with low or very low SES; for this reason, they should be widely included in national surveys and in representative studies on the prevalence of anemia, something that currently does not usually occur. We also assessed the effectiveness of nutritional intervention programs, observing a clear benefit, especially from those at the national level, as we discuss below.

A significant difference in the prevalence of anemia was found according to children’s age, the percentage of anemia being higher in preschool children than in school-age ones. The growth rate and nutritional requirements are high until 5 years of age [2,74], which makes young children more vulnerable to malnutrition and vitamin and mineral deficiencies. We also observed that the risk of iron deficiency anemia affected preschool children regardless of their SES, probably because the rapid growth typical of their age occurs in an unfavorable global environment that is not able to meet the child’s requirements. In relation to this, poor hygienic conditions, helminth infections, malaria, and the prolonged breastfeeding in replacement of a complete and adequate diet has been related with anemia in young children [2,75,76]. However, as children grow, the prevalence of anemia seems to drop off due to the rate of growth and the nutritional requirements slowing down. This was more visible in those school-age children of middle SES, where the social and environmental factors related to anemia could be more controlled. Accordingly, the benefits offered by regions with middle SES, such as increased access to medical care and deworming programs, improved hygienic conditions, and increased food security, could compensate for other factors and result in the difference between middle SES children and those living below the poverty line [2,77].

Although the risk of anemia is patterned by gender throughout the life course, women having a greater prevalence of anemia than men, this circumstance does not occur until puberty [2]. Conversely, anemia appears to be more common in boys than in girls [78,79] at early ages or there is simply not usually gender-related differences in young children [80,81,82], which agrees with our observations. Furthermore, we neither observed any differences between girls aged 9 to 12 years old and boys at these ages, contrary to the premise that menstrual blood losses could increase the risk of anemia in girls [2].

The nutritional transition that LAC has undergone during the last three decades and is still being experienced by some countries [83,84,85] could be the reason for the unexpected lack of effect of rurality on the prevalence of anemia, as has historically occurred. Although we observed that anemia was less frequent in urban than in rural areas, especially in school-age children (16.86%; 95%CI: 6.93, 26.80, and 25.87%; 95%CI: 6.50, 45.25, respectively), the association was not significant (*p* = 0.42). This suggested that despite the diet of children living in cities possibly being wider and more varied than in rural areas, it is increasingly based on processed foods, rich in sugars and fats, which do not contain a sufficient amount of iron and other vitamins and minerals. Along with sedentary lifestyle linked to urban environments, this rapid change in dietary patterns provides an obstacle when it comes to reducing anemia in cities, despite the greater availability of food.

In order to address the public health problem posed by anemia, several intervention programs and health policies have been implemented during the last two decades in LAC countries. After this systematic review, we observed that these actions tend to focus on young children as the most vulnerable population group and often neglect children over 5 years of age. This is something that should be corrected, having observed that school-age children are also at risk of anemia, especially those from low-income regions. As anemia is caused mainly by iron deficiency [1,3], the health strategies launched had a marked nutritional aspect. Food delivery, distribution of micronutrient powders and iron supplements, and fortification of staple foods are the most frequent form of nutritional intervention [2]. Costa Rica has been a pioneer in mass food fortification including wheat flour, maize flour, and liquid and powdered milk since 1999 [53], which could explain the low prevalence of anemia among Costa Rican children. According to our results, anemia was not a concern either, or it was simply a mild public health problem, for children in Chile and Argentina. It was likely a consequence of the national plans launched by the countries of the Southern Cone since 2000. In Chile, the National Complementary Feeding Program [52] was based on iron fortification of powdered milk, while Argentina implemented, in addition, fortification of wheat flour and iron supplementation for children under 2 years of age [3]. In addition to food fortification, Cuba [55] and the Dominic Republic [25] included food delivery in their national plans to improve the availability and diversity of food in the general population. In addition, Mexico [61] and Peru [69] implemented comprehensive plans involving health services, nutritional and health education, and even cash transfers. Good results were reported after these interventions on a larger scale; the prevalence of anemia was reduced from 32 to 26% in Cuba, from 44 to 17% and 26% in the Dominican Republic and Mexico, respectively, and even decreased by more than 40 percentage points in Peru. These achievements suggested, therefore, that not only nutritional deficiencies but the global social and household environment could determine the risk of anemia in children [72,77].

On the other hand, considering LAC regions, the Latin Caribbean and especially Haiti [60] lead the list in terms of the prevalence of anemia, representing a severe public health problem for young children. It should be noted that the Haitian government has never established a national plan to reduce anemia [86], and it has been documented that regional interventions with milk or complementary foods fortified with iron have generally not been successful, although some isolated positive results were found [71]. In a similar way, Jamaica [59], Panama [14], and some regions of Brazil [28,38,43,44,45,46,47,48,49] also exceeded 40% prevalence of anemia. However, there is no record of any nutrition intervention program in Jamaica, and, in the case of Brazil, some reports highlight the low coverage and inadequate compliance with the National Iron Supplement Program approved by the government in 2005 [87,88]. As to Panama, the National Plan for the Prevention and Control of Micronutrient Deficiencies was active between 2008 and 2015, but there are no data available to date on the observed effects on the prevalence of anemia [89].

Following the nutritional intervention programs, some countries continued to have high rates of anemia [60,65], while others reduced its prevalence significantly [52,67,70,71,72,73]. Although similar actions were carried out in Haiti [60,71], Brazil [72], Bolivia [70], Peru [65,68], Ecuador [67], Mexico [73], and Chile [52], their effectiveness seems not to be the same. The success or failure of health and nutritional interventions, especially in populations with low or very low SES, has been related with multiple causes, including coverage, participant compliance, monitoring, and quality in reporting results [2,90]. This could therefore explain our observation that, although some regional or local studies showed good results at the individual level, jointly, only those programs designed at a national level, with great coverage, well monitored, and extended over time had success in reducing the prevalence of anemia in the children of the LAC countries.

## 5. Conclusions

Anemia remains a public health problem for children in LAC countries, especially for children under 5 years old. The implementation, expansion, and good monitoring of nutritional intervention programs at the national level are needed to control anemia. They should be designed to address the direct and indirect determinants of anemia, according to the specific needs of children in each country.

## Figures and Tables

**Table 1:** Characteristics of studies that assessed preschool children (under 5 years).

Author	Year	Country	SES	Study Design	Sample Size (*n*)	Anemic Children (*n*)	Anemia		Quality of Studies
							%	95% CI	
CFNI/PAHO_Jamaica [59]	1998	Jamaica	Low	Cross–sectional	272	131	48.20	42.26–54.14	Fair
Neuman NA, et al. [43]	2000	Brazil	Middle	Cross–sectional	468	284	60.60	56.17–65.03	Good
Osório MM, et al. [44]	2001	Brazil	Very low	Cross–sectional	777	318	40.90	37.44–44.36	Good
Rivera JA, et al. [61]	2004	Mexico	Low	Cross–sectional	595	154	25.80	22.28–29.32	Good
Assis AM, et al. [45]	2004	Brazil	Middle	Cross–sectional	603	279	46.30	42.32–50.28	Good
Morais MB, et al. [46]	2005	Brazil	Very low	Cross–sectional	108	70	64.80	55.79–73.81	Fair
Ministry of health of Panama [14]	2006	Panama	Very low	National Survey	373	156	41.80	36.79–46.81	Fair
Brooker S, et al. [47]	2007	Brazil	Very low	Cross–sectional	139	44	31.65	23.92–39.38	Fair
EMMUS IV [17]	2007	Haiti	Low	National Survey	4142	2599	60.60	59.11–62.09	Fair
Duque X, et al. [31]	2007	Mexico	Middle	Cross–sectional *	4957	986	19.90	18.79–21.01	Good
Vieira AC, et al. [48]	2007	Brazil	Very low	Cross–sectional	153	85	55.60	47.73–63.47	Fair
SIVIN 2007 [21]	2008	Nicaragua	Very low	National Survey	1466	295	20.10	18.05–22.15	Good
PNDS 2006 [18]	2009	Brazil	Middle	National Survey	3455	721	20.86	19.51–22.21	Good
Durán P, et al. [29]	2009	Argentina	Middle	Cross–sectional *	30,514	5035	16.50	16.08–16.92	Good
GDSH 2009 [20]	2009	Guyana	Middle	National Survey	1349	316	23.40	21.14–25.66	Good
Oliveira CSM, et al. [49]	2011	Brazil	Middle	Cross–sectional	429	246	57.30	52.62–61.98	Good
Castro TG, et al. [50]	2011	Brazil	Middle	Cross–sectional	617	180	29.20	25.61–32.79	Good
Leal LP, et al. [34]	2011	Brazil	Middle	Cross–sectional	1403	476	33.92	31.44–36.40	Good
Cardoso MA, et al. [35]	2012	Brazil	Middle	Cross–sectional	526	111	21.10	17.61–24.59	Good
Rocha DS, et al. [36]	2012	Brazil	Middle	Cross–sectional	312	102	32.80	27.59–38.01	Good
Brito A, et al. [51]	2012	Chile	Middle	Cross–sectional	224	12	5.40	2.44–8.36	Fair
Huamán–Espino L, et al. [65]	2012	Peru	Very low	Cross–sectional	714	366	51.30	47.63–54.97	Good
ENSANUT-ECU 2012 [16]	2012	Ecuador	Middle	National Survey	1913	356	18.60	16.86–20.34	Fair
Oliveira AP, et al. [37]	2013	Brazil	Middle	Cross–sectional	980	363	37.00	33.98–40.02	Good
Silla L, et al. [38]	2013	Brazil	Middle	Cross–sectional	2186	992	45.40	43.31–47.49	Good
ENDESA 2011-2012 [19]	2013	Honduras	Very low	National Survey	10,681	3097	29.00	28.14–29.86	Good
Brito A, et al. [52]	2013	Chile	Very low	Cross–sectional	320	44	13.75	9.98–17.52	Good
Leite MS, et al. [28]	2013	Brazil	Very low	Cross–sectional *	5397	2763	51.20	49.87–52.53	Good
National Micronutrients Survey [24]	2014	Dominican Republic	Low	National Survey	772	217	28.10	24.93–31.27	Fair
ENS 2014 [23]	2014	El Salvador	Very low	National Survey	5862	1327	22.64	21.57–23.71	Good
Oliveira TSC, et al. [39]	2014	Brazil	Middle	Cross–sectional	373	143	38.30	33.37–43.23	Good
Sarmiento OL, et al. [27]	2014	Colombia	Middle	Cross–sectional *	7725	2124	27.50	26.50–28.50	Good
Saraiva BC, et al. [40]	2014	Brazil	Middle	Cross–sectional	692	109	15.70	12.99–18.41	Good
Pita GM, et al. [55]	2014	Cuba	Low	Cross–sectional	2204	573	26.00	24.17–27.83	Good
Vasconcelos PN, et al. [41]	2014	Brazil	Middle	Cross–sectional	646	135	20.90	17.76–24.04	Fair
Iannotti L, et al. [60]	2015	Haiti	Low	Cross–sectional	182	128	70.30	63.66–76.94	Good
Rodríguez–Zúñiga MJ, et al. [66]	2015	Peru	Low	Cross–sectional	473	137	29.00	24.91–33.09	Good
Martorell R, et al. [53]	2015	Costa Rica	Very low	Cross–sectional	403	16	4.00	2.09–5.91	Fair
ENDES 2016 [15]	2017	Peru	Low	National Survey	10,060	3350	33.30	32.38–34.22	Good
EDSA 2016 [26]	2017	Boliva	Low	National Survey	1526	819	53.70	51.20–56.20	Fair
ENSMI 2014-2015 [22]	2017	Guatemala	Low	National Survey	11,164	3617	32.40	31.53–33.27	Good
Robles BN, et al. [13]	2017	Dominica	Very low	Retrospective	635	123	19.30	16.23–22.37	Good
Barquero MS, et al. [54]	2018	Costa Rica	Very low	Cross–sectional	1291	111	8.60	7.07–10.13	Fair
Cruz-Góngora V, et al. [30]	2018	Mexico	Middle	Cross–sectional *	9094	2446	26.90	25.99–27.81	Good
Assandri E, et al. [58]	2018	Uruguay	Middle	Cross–sectional	136	45	33.00	25.10–40.90	Fair

SES: socioeconomical status; * data from national survey.

**Table 2:** Characteristics of studies that assessed schoolchildren (5-12 years).

Author	Year	Country	SES	Study Design	Sample Size (*n*)	Anemic Children (*n*)	Anemia		Quality of Studies
							%	95% CI	
Quizhpe E, et al. [57]	2003	Ecuador	Middle	Cross–sectional	592	98	16.60	13.60–19.60	Good
Monárrez–Espino J, et al. [62]	2004	Mexico	Very low	Cross–sectional	331	43	13.00	9.38–16.62	Fair
Reboso J, et al. [56]	2005	Cuba	Low	Cross–sectional	100	22	22.00	13.88–30.12	Fair
Ministry of health of Panama [14]	2006	Panama	Very low	National Survey	788	50	6.30	4.60–8.00	Fair
Winocur D, et al. [32]	2007	Argentina	Middle	Cross–sectional	323	8	2.50	0.80–4.20	Good
Brooker S, et al. [47]	2007	Brazil	Very low	Cross–sectional	301	32	10.63	7.15–14.11	Fair
ENSANUT-ECU 2012 [16]	2012	Ecuador	Middle	National Survey	4443	156	3.50	2.96–4.04	Fair
Cardoso MA, et al. [35]	2012	Brazil	Middle	Cross–sectional	585	40	6.80	4.76–8.84	Good
ENM 2012 [25]	2013	Dominican Republic	Low	National Survey	654	119	18.20	15.24–21.16	Fair
Sarmiento OL, et al. [27]	2014	Colombia	Middle	Cross–sectional *	7906	640	8.10	7.50–8.70	Good
Rodríguez–Zúñiga MJ, et al. [66]	2015	Peru	Low	Cross–sectional	1.438	905	62.90	60.40–65.40	Good
Cabada MN, et al. [63]	2015	Peru	Very low	Cross–sectional	192	70	36.50	29.69–43.31	Fair
Iannotti L, et al. [60]	2015	Haiti	Low	Cross–sectional	753	509	67.59	64.25–70.93	Good
Aparco JP, et al. [64]	2016	Peru	Low	Cross–sectional	808	96	11.90	9.67–14.13	Fair
Ferreira HS, et al. [42]	2016	Brazil	Middle	Cross–sectional	1547	144	9.30	7.85–10.75	Good
Barquero MS, et al. [54]	2018	Costa Rica	Very low	Cross–sectional	912	55	6.00	4.46–7.54	Fair
Cruz-Góngora V, et al. [30]	2018	Mexico	Middle	Cross–sectional *	15,993	1999	12.50	11.99–13.01	Good
Lázaro L, et al. [33]	2018	Argentina	Middle	Cross–sectional	362	16	4.40	2.29–6.51	Fair

SES: socioeconomical status; * data from national survey.
